# Agile, on-demand wastewater surveillance of virus infections to support pandemic and outbreak response in Rotterdam-Rijnmond, the Netherlands, 2020 to 2022

**DOI:** 10.2807/1560-7917.ES.2024.29.47.2400055

**Published:** 2024-11-21

**Authors:** Emma Besijn, Jane Whelan, Paul Bijkerk, Gregorius J Sips, Jeroen Langeveld, Ray W Izquierdo-Lara, Elvira van Baarle, Remy Schilperoort, Marion P G Koopmans, Miranda de Graaf, Gertjan Medema, Ewout Fanoy

**Affiliations:** 1 Public Health Service Rotterdam-Rijnmond, Rotterdam, The Netherlands; 2 Partners for Urban Water, Nijmegen, The Netherlands; 3 Sanitary Engineering, Delft University of Technology, Delft, The Netherlands; 4 Department of Viroscience, Erasmus University Medical Center, Rotterdam, The Netherlands; 5 KWR Water Research Institute, Nieuwegein, The Netherlands; * These authors contributed equally to this work and share first authorship.; ** These authors contributed equally to this work and share last authorship.

**Keywords:** Wastewater, Cities, SARS-CoV-2, Disease outbreaks, Mpox virus, Students, passive samplers

## Abstract

**Background:**

Wastewater surveillance may support early and comprehensive detection of infectious diseases’ community transmission, particularly in settings where other health surveillance systems provide biased or limited information. Amid the SARS-CoV-2 pandemic, deploying passive samplers to monitor targeted populations gained importance. Evaluation of the added public health value of this approach in the field can support its broader adoption.

**Aim:**

We aimed to assess the feasibility and utility of on-demand wastewater surveillance, employing passive samplers, for SARS-CoV-2 and monkeypox virus (MPXV) in small/targeted populations, also considering ethical aspects.

**Methods:**

Pilot case studies in the Rotterdam-Rijnmond region were used for a systematic assessment of the feasibility and utility of wastewater monitoring of SARS-CoV-2 (variants) and MPXV using passive sampling. Each case study was instigated by actual questions from the Public Health Service about disease transmission.

**Results:**

Case study results demonstrated the feasibility and utility of on-demand wastewater surveillance with successful identification of a local peak in SARS-CoV-2 transmission, early detection of wider Omicron variant transmission after the first case was reported, as well as indication of no emerging local MPXV transmission. Ethical considerations led to the abandonment of one case study involving a displaced population.

**Conclusions:**

The study confirms the feasibility and utility of passive sampling for real-time infectious disease surveillance, at desired spatiotemporal resolution. Ethical concerns and operational challenges were identified, highlighting the need for early stakeholder engagement and ethical guideline adherence. The method could be used to study under-surveyed populations and be extended beyond SARS-CoV-2 and MPXV to other pathogens.

**Key public health message**

**What did you want to address in this study and why?**

In a Dutch urban region, case studies in 2020−2022 tested if local community-level wastewater surveillance could provide comprehensive, real-time infectious disease transmission data upon acute demand from a public health (PH) service. Passive samplers for SARS-CoV-2 and monkeypox virus (MPXV) were deployed in small wastewater catchment areas to assess this method’s feasibility, utility, adaptability, strengths and limitations in informing PH actions.

**What have we learnt from this study?**

A large increase in SARS-CoV-2 RNA concentration in wastewater was timely identified in a locality, triggering the PH service to further inform people about the virus there and offer testing for it. Omicron detection in wastewater where the first Omicron case was reported confirmed this variant’s wider circulation in the community. For MPXV, wastewater data suggested no further local transmission. One case study highlighted ethical constraints.

**What are the implications of your findings for public health?**

The case studies suggested that on-demand wastewater surveillance using passive samplers can be a valuable and agile tool for tracking the spread of SARS-CoV-2 and MPXV at community level. The method's ability to detect emerging SARS-CoV-2 variants and early transmission patterns allowed to improve the situational awareness of the PH service and guided targeted PH interventions. Ethics and stakeholder engagement are important for the application.

## Introduction

Wastewater surveillance is the process of monitoring wastewater for pathogens in human communities. Pathogens that are excreted in bodily fluids and excrements may be detectable in domestic wastewater, sometimes before infected individuals are symptomatic [1,2]. During the COVID-19 pandemic, an objective of wastewater surveillance was to identify COVID-19 outbreaks early. Another aim was to obtain more insight into the circulation of severe acute respiratory syndrome coronavirus 2 (SARS-CoV-2), the virus causing COVID-19. This included studying trends of SARS-CoV-2 RNA concentrations in wastewater over time, investigating the occurrence and spread of virus variants of concern (VOC) or variants of interest (VOI) [2], as well as surveying changes in the geographical and spatial distributions of SARS-CoV-2 infections coinciding with different socio-demographic contexts.

Detection of genetic material in wastewater using reverse-transcription quantitative PCR (RT-qPCR), allows for surveillance of circulating viruses in the population and a more complete representation of the bottom of the surveillance pyramid, including asymptomatic and mildly symptomatic infections that are not detected in conventional syndromic or clinical surveillance. While the added value of wastewater surveillance targeting a pathogen depends on the clinical profile of the infection with this pathogen (such as incubation time, severe vs mild vs no symptoms while infected, virus shedding etc.), it has been clearly demonstrated for trend-monitoring of SARS-CoV-2 [3] and as an early warning tool for poliovirus [4]. The potential added value of wastewater surveillance has also been discussed for other infections, including among others, those with monkeypox virus (MPXV), influenza virus and respiratory syncytial virus [5].

Wastewater sampling has typically been conducted at intakes of large wastewater treatment plants, using (costly) auto-sampling equipment. However, this approach is not easily applicable to small catchment areas, such as sewer manholes. Yet, such small-scale sampling could offer more rapid and agile deployment and high spatial resolution measurements, facilitating public health action aimed at specific community or facility levels, like long-term care facilities or university campuses [6]. Recently, small, cheap, easily deployable passive sampler units have been developed and validated for SARS-CoV-2 detection, making rapid ‘on-demand’ surveillance possible at such localised scales [6,7]. Their application in a quality-assured, standardised manner for such surveillance has nevertheless not been widely evaluated, nor has the method’s feasibility, timeliness, and reliability to yield results that are interpretable and actionable for public health. This lack of evaluation potentially limits incentives for public health authorities to use or rely on this ‘on-demand’ surveillance.

A regional Public Health Service (PHS) in the Netherlands commenced with on-demand wastewater surveillance to evaluate its feasibility for SARS-CoV-2 and later for MPXV in public health practice and its added value compared with traditional surveillance to inform targeted control measures to limit the spread of infectious disease. In this article, we consider the feasibility and utility, as well as the strengths and limitations of the technique through a series of pilot studies conducted in the Public Health Service Rotterdam-Rijnmond (PHS-RR) region. We also discuss the direction of future development of the tools for public health action.

## Methods

### Study setting

Rotterdam-Rijnmond is a densely populated urban region hosting 1.3 million people of considerable ethnical diversity. The Port of Rotterdam, the largest port in Europe, adds to the flow of diverse people to the city, and there is wide variation in socioeconomic, educational and health status in the region [8].

### Pilot studies’ objectives

Six pilot studies were conducted, selected based on public health needs as identified by the PHS. The objectives of the case studies were as follows: to determine the feasibility and reliability of wastewater surveillance using passive sampling for the detection of SARS-CoV-2 in wastewater (case study 1, October 2021); to evaluate community transmission around the first reported case of the Omicron variant in the service area of the Rotterdam Rijnmond public health agency (case study 2, December 2021); to detect and monitor viral spread among groups of interest based on their risk profile for SARS-CoV-2 infection (case studies 3−5), with case study 3 (May 2022) focusing on displaced populations, case study 4 (September–November 2022) on university students and case study 5 (September–November 2022) on people with different vaccination rates; and to identify if there was evidence of community MPXV spread after the diagnosis of the first case of mpox (case study 6, June–July 2022).

### Wastewater passive sampling methodology

A passive sampler is a plastic porous container with absorption materials inside, such as the cotton tip used for this study. Two types of passive samplers shown in Supplementary Figure S.1 in the Supplementary Information were used in these pilot studies: a torpedo (larger, for application in pumping stations, and pensive (smaller, for application in sewer lines) sampler, both obtained from D. McCarthy, Monash University, Australia [7].

The samplers were installed at the desired locations (e.g. sewage treatment plant inlet, sewer pipe or pumping station) using ropes. After being in place for 24 to 72 hours, the passive samplers were collected and taken to the laboratory. The plastic casing was removed, and the cotton swab that was inside the casing was immersed in nucleic acid extraction buffer (Nuclisens, Biomerieux, Amersfoort, the Netherlands). Nucleic acids (RNA and DNA) were extracted from the material in the cotton swabs in combination with the semi-automated KingFisher mL (Thermo Scientific, Bleiswijk, the Netherlands) purification system and tested for the presence of SARS-CoV-2 RNA (N2 gene) and for CrAssphage DNA using RT-qPCR and qPCR respectively, as prior described [3,9]. Viral concentration was quantified in genome copies per mL (GC/mL). Next generation sequencing (NGS) and data analysis were conducted as described previously [10]. Presence of signature mutations of specific VOC’s was determined using droplet digital (dd) RT-PCR conducted on 5 µL of nucleic acid extract using G339D and N856K assays [9]. ddPCR was also used for MPXV DNA [11], using the dEXD51818561 research assay of BioRad, with positive controls from the gBlock described by BioRad, as well as MPXV DNA provided by Erasmus University Medical Center. In addition, qPCRs were conducted for both orthopoxvirus [12] and MPXV [13] on an aliquot of the nucleic acid extracts of the passive samplers. 

Sampling locations were selected based on the specific objectives of the pilot while also taking account of the safety and accessibility of the test locations. The information on the local sewer network (map, location and diameter of sewer pipes, manholes, pumping stations, wastewater treatment systems, combined or separate sewer) was obtained from the municipality and site visits provided information on accessibility, (road) safety, fouling and ability to deploy passive samplers, sewage flow, water depth and flow direction at the sampling sites. Sewer networks in the Netherlands are typically mazed due to the low gradient and, consequently, flow directions are not always clear from sewer maps and need to be confirmed by site visits.

### Test validity and quality control

To distinguish between actual variations in SARS-CoV-2 levels and apparent variations caused by differences in the dilution of the sewage due to precipitation, extraneous waters or industrial wastewater or different amounts of material captured by the passive sampler, the results from the samplers were normalised based on levels of CrAssphage, by dividing the number of gene copies of SARS-CoV-2 by those of CrAssphage for each sample [14]. CrAssphage is a bacteriophage uniquely present in high concentrations in human faeces. The results of the separate measurements of SARS-CoV-2 RNA (N2 gene) by RT-qPCR or MPXV DNA by ddPCR and the CrAssphage DNA by qPCR can be combined to allow for a quantitative comparison of the results between different measuring points. Laboratory analyses on each sampler material were conducted in duplicate, also including positive and negative controls. Mouse hepatitis virus (MHV), an animal coronavirus, was added to monitor inhibition of the extraction and RT-qPCR. 

Further quality control was based on photos and videos of the passive samplers before and after installation. Additionally, measurements were also taken at two reference locations to investigate the comparability between the 'normal' 24-hour volume-proportional sampling by automatic sampling cabinets and passive samplers. Results were evaluated at the end of each measurement week and at the conclusion of the pilot.

### Evaluation of on-demand wastewater passive sampling surveillance

For each pilot study, specific attributes of a quality public health surveillance system [15] were evaluated (Table 1). Attributes focused on quantitative aspects that reflected the overall reliability of the methods used, including how well the presence of a virus or its variants observed in the targeted population was reflected in the wastewater monitoring data (sensitivity), to what extent differential viral distribution between communities or viral distribution by place and time was mirrored by the wastewater surveillance (representativeness), how adequately the wastewater sampling covered the targeted community (completeness), the percentage of passive samplers recovered and yielding adequate results and how the molecular findings compared with defined controls (validity) and the timeliness in days to get results from the wastewater monitoring. Other attributes were more qualitative in nature, evaluating the acceptability within communities, the feasibility and ease of implementation (simplicity), the flexibility based on changing demands of the public health agency, the consistency in different populations and their sewer catchments and the usefulness in informing public health action and policy. Each attribute was operationalised into study outcomes and endpoints as in Table 1.

**Table 1:** Quality attributes of wastewater surveillance that were evaluated during pilot case studies conducted in Rotterdam-Rijnmond, the Netherlands, 2020–2022 (n = 5 case studies)^a^

Attribute	Outcome	Endpoint
Sensitivity	Detection of SARS-CoV-2 (including VOC) or MPXV.	A positive signal for SARS-CoV-2 and VOC or MPXV in the target area with known case(s) can be obtained by the on-demand wastewater surveillance; positivity of passive samples for SARS-CoV-2 can be confirmed as similar to the positivity of 24-hour volume proportional samples, which are used as a reference.
Representativeness	Occurrence of a reported infectious pathogen and its distribution in the population by time and place is reflected in the wastewater of that same population.	Differential viral circulation at community level observed in reported cases is mirrored by the signals in wastewater in carefully matched wastewater samples in the same geographic area, neighbourhood, or institution or over time.
Completeness	Ability to monitor the complete target community during the monitoring period.	Targeted neighbourhoods or communities can be adequately covered with passive samplers deployed at the sewer network servicing this population.
Validity	Results are trustworthy.	In the laboratory, positive and extraction controls yield a positive result, PCR blanks and negative controls yield a negative result. Percentage of placed passive samplers that are recovered and yield adequate results.
Timeliness	Rapid availability of results for implementation of timely virus spread control measures.	Three time-intervals in days are considered: (i) between agreeing on the pilot objectives among stakeholders and placement of passive samplers; (ii) between retrieval of passive samplers and receipt of laboratory test results; (iii) between an increase in the proportion of positive reported test from public health PCR testing and increase in viral circulation in wastewater.
Acceptability	Willingness of organisations or people to cooperate.	Communities affected agree or not to participate.
Simplicity	Feasibility and ease of implementation and operation.	Pilot studies can be conducted in practice within available resources (personnel, time, cost).
Flexibility	Quick implementation at various locations and potential to adapt when information needs change.	Proportion of desired locations that are successfully sampled in the desired period.
Consistency	Virus circulation is captured systematically based on circulation in the population.	Similar circulation in different populations is mirrored by results of the passive water surveillance of their respective sewer catchments.
Usefulness	Results can inform decision-making and public health action.	Was public health action implemented based on the results? (yes/no)

MPXV: mpox virus; SARS-CoV-2: severe acute respiratory syndrome coronavirus 2; VOC: variant of concern.

^a^ A total of six studies were initially envisaged but one study involving a displaced population had to be cancelled based on ethical considerations.

## Results

Details of the characteristics of each pilot, including specific objectives, hypotheses and some assessed attributes of the wastewater surveillance (i.e. sensitivity and usefulness) are summarised in Table 2. An itemised evaluation of all the attributes for each case study is presented in Supplementary Table S.1.

**Table 2:** Details of pilot case studies using passive sampling for wastewater surveillance in Rotterdam-Rijnmond, the Netherlands, 2020–2022 (n = 6 studies)

Cases study (period)	Objective/hypothesis to test	Sensitivity	Usefulness
**1** (6–29 Oct 2021)	Testing, if detecting SARS-CoV-2 in wastewater in different community contexts, with passive samplers is feasible and reliable.	In a locality of Rotterdam-Rijnmond large coinciding peaks were observed both in COVID-19 case numbers detected through COVID-19 testing centres in the last week of October and in SARS-CoV-2 concentration in wastewater tested on 27 October 2021. Similar positivity rate and concentrations of SARS-CoV-2 RNA in 24-hour volume-proportional water samples (taken with an autosampler) and passive samples; good correlation between the virus concentrations observed, and similar concentration-patterns over time observed in both sampler types.	The result triggered the public health service to focus information campaigns and further testing in the locality.
**2** (7–30 Dec 2021)	Evaluating community transmission after the first case of the Omicron variant was identified in the Rotterdam-Rijnmond region in late November 2021.	Higher concentrations of SARS-CoV-2 were detected in the first sampling week (7, 8 and 9 December) compared with the second sampling week (28, 29 and 30 December 2021). No Omicron was identified in the first sampling week (ddPCR and sequencing) but its presence was confirmed in passive samples of 28, 29 and 30 December 2021 taken in the hamlet where the first case of Omicron had been detected by surveillance in people, as well as in a sewer pumping station and a wastewater treatment plant located downstream^a^. Numerical values of the Omicron proportion relative to overall SARS-CoV-2 (mostly Delta) were variable per sampling day and location, probably due to relatively low proportions of Omicron (average: 8.0%; standard deviation: 11.7%). The passive samplers and the 24-hour volume-proportional samplers showed comparable results as illustrated in Supplementary Figure S.4.	The occurrence of Omicron VOC at each of the wastewater sampling sites, indicated that this VOC was broadly present within the community. Authorities thus improved real-time situational awareness in the community and informed the medical and PH community about local ongoing transmission, while also concluding that quarantining and contact tracing were no longer appropriate to restrict the spread of this VOC.
**3** (7 Apr–15 May 2022)	Early warning of viral introduction in displaced populations.	Due to potential stigmatisation of this population and the complexity of obtaining consent from all involved the case study was cancelled.	
**4** (7 Sep–14 Nov 2022)	Determining if student houses are hotspots for the introduction of SARS-CoV-2 in Rotterdam after the summer holidays.	SARS-CoV-2 was detected at each student home, but at different positivity rates and (normalised) concentrations. CrAssphage indicated that similar amounts of human faeces were sampled in each sample. It was not possible to take 24-hour volume proportional samples^b^.	Variability of the observed concentration in sewers from the student houses hindered firm conclusions about the students being 'introducers' of SARS-CoV-2 after the summer holidays. These findings did not call for specific public health action to further contain SARS-CoV-2 spread in/from student houses.
**5** (7 Sep–14 Nov 2022)	Determining if neighbourhoods with lower vaccination coverage have more current SARS-CoV-2 infections than neighbourhoods with higher coverage.	Virus circulation was shown among all the populations measured through passive sampling of the community’s wastewater. Distribution of viral circulation was equal in most neighbourhoods, except for one of the neighbourhoods with high vaccine coverage, which had a high amount of SARS-CoV-2 present in October 2022. There was no explanation for why this area had higher virus-circulation. It was not possible to take 24-hour volume proportional samples^b^.	Differences in vaccination coverage were not very large, data on acquired immunity due to prior infections were not complete and information on frequency and diversity of contacts of the communities was not available. Due to limitations, the study findings as such could not support additional public health measures to stop the spread of SARS-CoV-2 at local level.
**6** (27 Jun–1 Jul 2022)	Detecting local transmission of MPXV.	In the street section of a person with MPXV, the virus DNA was detected in the passive samples of the wastewater, in 20–80 copies per passive sample, both through the Orthopox qPCR and MPXV qPCR and ddPCR assay. It was not possible to take 24-hour volume proportional samples^b^.	The presence in the wastewater in the street of the case, but not in the surrounding area which was also studied by on-demand passive surveillance, showed proof-of-concept of MPXV detection in wastewater and indicated that there was no further local spread of MPXV. No further/additional public health action was needed.

ddPCR: droplet digital PCR; MPXV: mpox virus; PH: public health; qPCR: quantitative PCR; SARS-CoV-2: severe acute respiratory syndrome coronavirus 2; VOC: variant of concern.

^a^ Samples processed from passive samplers could be sequenced just as well as from sewage water samples. Omicron was identified through ddPCR and sequencing.

^b^ Since case studies 1 and 3 showed comparable results between the two, we considered that the sensitivity of the passive samplers was demonstrated convincingly in the earlier case studies where sampling with both methods concurrently was a possibility.

**Figure 1:**
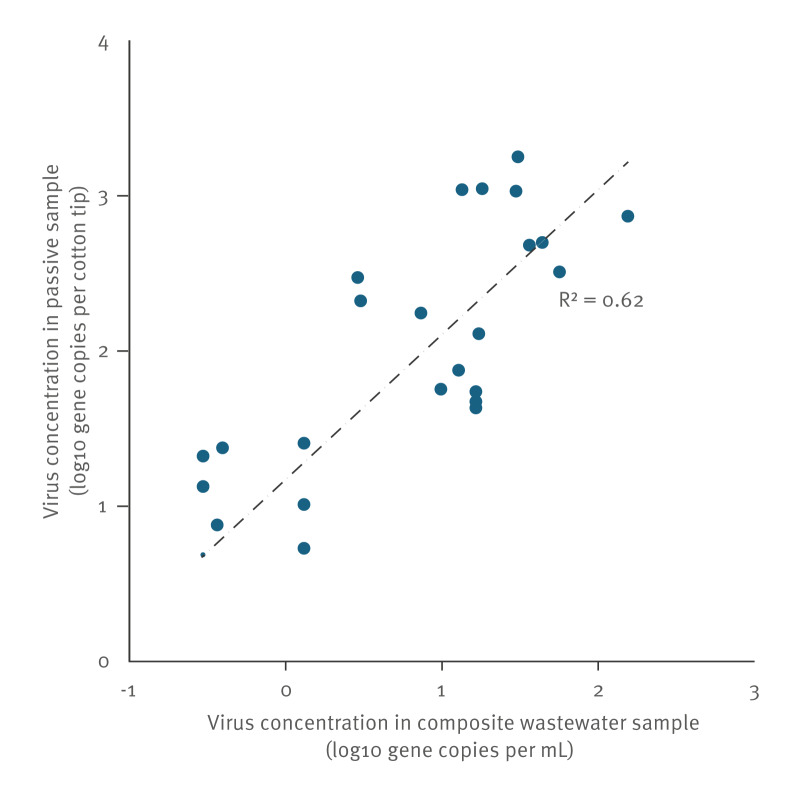
Correlation between SARS-CoV-2 concentrations from 24-hour passive samplers (y-axis) and the 24-hour volume-proportional wastewater samples for virus capture from wastewater (x-axis)

Case study 1 from 6 to 29 October 2021 ran for 4 weeks in three different areas of the city, where wastewater was sampled with passive samplers twice a week. This case study showed that deployment of passive samplers in the sewer via manholes and at pumping stations was feasible. The monitoring sites served population sizes ranging from ca 7,000 to 21,000 inhabitants. The CrAssphage concentrations that were recovered showed that 72 of 76 passive samplers consistently picked up human faecal material. The four passive samplers that picked up very low amounts of human faecal material were either blocked by deposition of wipes and other materials on the outside of the sampler or were moved outside of the wastewater flow (Figure S.1). As indicated in Figure 1, the correlation between the concentrations of the virus captured from wastewater by passive samplers that were deployed for 24 hours at the pumping stations and those of the composite water samples collected over the same 24 hours at the same pumping stations (n = 24) was good (Pearson’s R^2^ = 0.62). Normalisation of the passive sampler results with CrAssphage reduced the correlation (Figure S.2). Comparing the patterns in the SARS-CoV-2 concentration observed in wastewater from different communities in the different city areas, with the patterns in reported cases in these areas, found peaks in reported cases reflecting peaks in wastewater concentration of SARS-CoV-2. Supplementary Figure S.3 shows concurrent peaks in reported cases and wastewater in city area 3 in the week of 11−17 October and in city area 2 in the week of 25−31 October. In contrast, a high incidence of reported cases in city area 3 in the week of 18−25 October was not reflected by a high concentration in wastewater. Smaller trends in virus circulation in the community were more difficult to observe due to the variability in both wastewater and case data. An estimation of the timeliness was made based on the experience in this case study. Provided that the monitoring objectives are clear and the information on the sewer network is already available, the minimum time between start and first result was 3 days. This would be in a situation where all information and stakeholders are available, and the objectives are (pre)planned.

Case study 2 from 7 to 30 December 2021 investigated community transmission after the first case of the Omicron variant was identified in the region. Samples (n = 26) were taken at a sewer pumping station serving the hamlet where the Omicron index case resided (ca 1,000 inhabitants), as well as a sewer pumping station in a town 6 km downstream (ca 5,000 inhabitants) and a wastewater treatment plant 5 km downstream of the town (serving ca 15,000 inhabitants). Like in the first case study, the passive samplers and the 24-hour volume-proportional samplers showed comparable results, as illustrated in Supplementary Figure S.4. All samples yielded valid results. Elevated levels of SARS-CoV-2, but not Omicron, were initially detected in the pumping station of the hamlet of the index case. Subsequently, the Omicron variant was successfully detected in wastewater, not only at the hamlet, but also downstream at the pumping station and the wastewater treatment plant of the wider community. The proportion of Omicron was generally low (average 8.0%; standard deviation 11.7%; n = 23) but was not lower in the samples from the larger population. This indicated that, despite the isolation measures rapidly instituted for the case, wider transmission in the local area was already ongoing and further quarantine and contact tracing measures would thus no longer be successful to contain onward spread. In this case study, we aimed to be as timely as possible, achieving a time of 6 days between the decision to start sampling at a specific location and first result of Omicron assays.

Case study 3 study was abandoned due to ethical considerations and therefore no results are reported. The population was regarded as both traumatised and sensitive to stigmatisation. In addition, the housing situation involved additional parties that were reluctant to participate in this case study. It was therefore decided to not pursue this case study.

**Figure 2:**
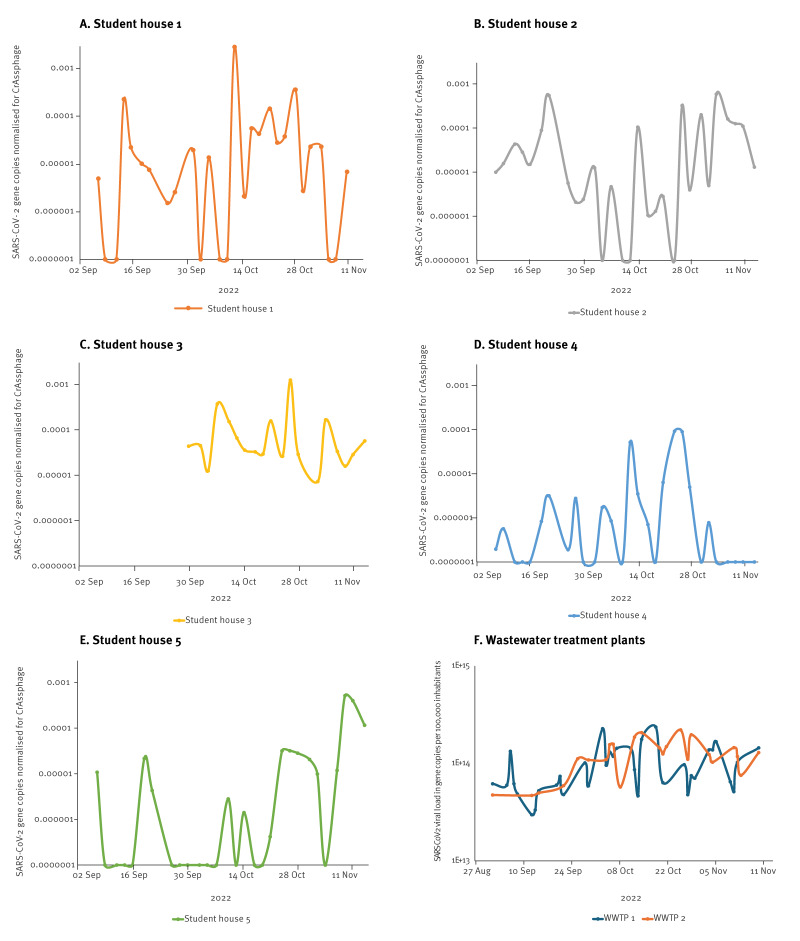
Normalised SARS-CoV-2 concentration in passive samplers at student residences (case study 4) and monitoring data from the Rotterdam wastewater treatment plant obtained from the national sewage surveillance at RIVM, Rotterdam-Rijnmond, the Netherlands, September 2022–November 2022

RIVM: Rijksinstituut voor Volksgezondheid en Milieu (National Institute for Public Health and the Environment); WWTP: wastewater treatment plant.

Case study 4 from 7 September to 14 November 2022, tested the hypothesis that students might be associated with the introduction of SARS-CoV-2 to their local communities, picking up the infection during summer holidays abroad, as was suggested after the summer holiday of 2020 [16]. A sampling campaign of student houses was preplanned during the summer (2 months), including selection of student houses and corresponding sampling sites. Sampling was started in the first week of the academic year. Five student-houses, inhabited by ca 220–350 students each, were sampled 2–3 times (48 or 72 hours) per week with passive samplers (n = 20–30 per student house). In total 140 samples were taken and 134 yielded a valid result. The samples of a week were analysed as one batch, making the time-to-result 1–5 days. Normalised SARS-CoV-2 concentrations in wastewater from the student houses were highly variable (Figure 2). Some houses, such as house 3, were consistently positive with relatively high (normalised) concentrations, while other houses, like house 4, fluctuated from negative to positive over time, with concentrations lower than the SARS-CoV-2 levels in wastewater from the larger catchment areas servicing Rotterdam. Overall, there was no consistent evidence of earlier or higher transmission in the student houses compared with the general population and the hypothesis was rejected (Figure 2).

Case study 5, from 7 September to 14 November 2022, sought to explore the relationship between vaccination coverage and SARS-CoV-2 circulation by analysing wastewater concentrations in two neighbourhoods with higher vaccination coverage (> 60% 1^st^ vaccine, > 45% 3^rd^ vaccine [8]) than three other neighbourhoods (< 45% 1^st^ vaccine, < 30% 3^rd^ vaccine [8]). The vaccination coverage was assessed in the month before the start of the sampling campaign. Neighbourhood sizes ranged from 1,300 to 29,100 inhabitants and these were monitored, depending on the sewage network at one to three sampling sites (n = 8 sampling sites in total). Each sampling site was investigated with 2–3 passive samples per week (i.e. 29–30 samples per sampling site over the study period). Of 238 samples in total, 229 gave a valid result. The samples of a week were analysed as one batch, making the time-to-result 1–5 days. Initially, our hypothesis was that a low vaccination rate would be associated with higher transmission since immunity reduces transmission of the virus [17]. However, our findings showed similar normalised SARS-CoV-2 concentrations in wastewater of the different areas, as illustrated in Supplementary Figure S.5, and one area with high vaccination coverage even had a relatively high amount of SARS-CoV-2 in wastewater in October 2022. The similar normalised SARS-CoV-2 concentrations in the area with high versus low vaccination rate indicated that viral shedding in these areas was similar, regardless of the differences in vaccination coverage in the examined areas. Sequencing data of the wastewater samples showed that the dominant VOC at that time was BQ1, as shown in Supplementary Figure S.6, which aligned with the prevalent VOC seen in clinical samples in the Netherlands in the autumn of 2022 [18].

In the case study 6 from 27 June to 1 July 2022, when the introduction of MPXV in the PHS-RR region by a traveller was reported, the question was whether there was wider transmission than the reported cases. Dedicated passive samplers were deployed in the sewer at the end of the street section where a documented person with MPXV resided and in two neighbouring areas. The first series of passive samplers were installed within 2 days of receipt of the request. Over the course of 1 week, three successive passive samplers collected the wastewater from a few hundred residents, each for 48 or 72 hours. All samples were valid. The samples of this week were analysed as one batch, making the time-to-result 1−5 days. MPXV presence was detected in the passive samples through a combination of qPCR assays for orthopoxvirus and MPXV assays and was further confirmed by additional ddPCR assay for West African MPXV. This demonstrated that MPXV was detectable in wastewater using passive samplers downstream of a reported case. The same methodology was repeated for a second person with MPXV, in another street in a different city area, yielding similar results (data not shown). 

## Discussion

We evaluated the attributes of a quality surveillance system in six pilot studies that were conducted during real-time, community-level infectious disease transmission events of SARS-CoV-2 and MPXV in the Rotterdam-Rijnmond region of the Netherlands. While one study had to be cancelled, for the remainder, once the aim and objectives of sampling were agreed between the PHS and key stakeholders, the underground sewage catchment areas were rapidly aligned with the above-ground location, and deployment of passive samplers was feasible at the various locations and in the contexts selected, including street-level communities, neighbourhoods and student residences. Passive samplers were generally installed within a day after the request was made by the public health service for acute case studies (1, 2 and 6) and on defined days for pre-planned case studies (4 and 5) and although some passive samplers were initially lost or yielded inadequate results due to clogging or movement outside the wastewater flow, adjustments to the attachment process proved successful (data not shown). Sampler losses were minimal and the feasibility of the method at a practical level was confirmed. 

In terms of quality assurance, sample results were only regarded valid when positive, negative and inhibition controls showed valid results, technical duplicate analyses showed comparable results and CrAssphage indicated that human faecal material was captured by the passive sampler. A key element of passive sampling is the need to relate the concentration of the target virus to the amount of human faecal wastewater monitored. CrAssphage normalisation was conducted for SARS-CoV-2 and passive samples yielded comparable results to 24-hour volume-proportional composite wastewater samples from pumping stations, further confirming the sensitivity of the monitoring and normalisation process, as also shown by Schang et al. [6], who used pepper mild mottle virus as faecal normaliser. Differences in CrAssphage shedding rates may lead to higher uncertainty in the normalisation as the population that is captured by the passive sampler becomes smaller [14].

The results from wastewater testing were considered timely, as the first results (i.e. detection of the virus by PCR) were available within 6 days of deciding to commence sampling at a specific location. Sequencing results followed within 14 days. Consequently, once the PHS identifies a need for targeted surveillance in a specific area, actionable data can be obtained within a week. This timeframe is considered sufficiently fast to initiate an effective response in standard practice.

In each case study, results from wastewater monitoring were interpreted in the context of wider public health surveillance systems. When comparing wastewater markers and reported cases in the same neighbourhood, both datasets exhibited concurrent patterns, as illustrated in Supplementary Figure S.3, indicating a good relationship between the two measures, as reported by Pico-Tomas [19].

A large increase in SARS-CoV-2 RNA concentration in one of the neighbourhoods was detected in a timely fashion (Figure S.3, city area 2), consistent with a large increase in reported cases in the same neighbourhood. This signal triggered the PHS to deploy additional mobile testing units to the area, and targeted information campaigns to intensify awareness on testing and tracing, the need for personal protective measures, the importance of vaccination, and hygiene and isolation and quarantine measures. Other successes included the early detection of the Omicron variant in the community sewers adjacent to the location where the first imported case was diagnosed in Rotterdam-Rijnmond. Evidence that community circulation was already ongoing allowed authorities to improve real-time situational awareness in the community and to inform stakeholders about local ongoing transmission, while also concluding that a ramping up of control measures would no longer be timely and effective.

In contrast, while MPXV was detected in the wastewater downstream of the home of a reported case, no detection was observed in surrounding areas, indicating no further local transmission. In this research effort, the selection of the sewer in the street of a known case was done to determine if MPXV was detectable in passive wastewater samples, rather than suggesting this as a common site for MPXV monitoring.

Sampling at student houses did not show high or early circulation of SARS-CoV-2 after the 2022 summer holidays. In the autumn of 2022 SARS-CoV-2 was detected in similar concentrations in wastewater of city areas with either higher or lower vaccination coverage. This observation is interesting against the background that immunity reduces transmission of the virus. Other observations on potential differences in these communities that may have affected SARS-CoV-2 transmission, such as the frequency and diversity of contacts [17], or acquired immunity due to previous infections with SARS-CoV-2, were difficult to make. This was because it was not possible to get an exact match between the population served by the sampled sewer network and the population for which vaccination or demographic data were reported in the available reporting systems [8]. Generally, the resolution of the vaccination rate or demographic data was lower than that of the sewer monitoring data. This makes conclusions from this case study difficult to draw.

A clear advantage of passive sampling over other forms of wastewater sampling is that it provides a high-level spatial and temporal resolution, allowing to study small or localised populations and, for example, locations that may be underserved by healthcare services or where residents do not otherwise access services. This study showed wastewater sampling can be done at the level of a student house with 220−350 residents. On the other hand, zooming in on small populations may pose certain ethical and operational challenges [20], which was reflected in two of our case studies. In case study 3, cooperation was sought to survey wastewater at locations where a displaced population was housed but local stakeholders did not agree, arguing that detection of SARS-CoV-2 in wastewater from the residential sites and related control measures such as lockdowns, isolation and/or quarantine, could lead to further stigma and trauma for residents. This pilot study was subsequently abandoned. In case study 5, it appeared that the rope anchoring the sampler inside the manhole had been cut. The context in which this occurred was unknown, but it may have indicated resistance among the local population to wastewater surveillance. 

Sewage surveillance in the Netherlands complies with the legal frameworks for privacy, and independent assessment at national level has confirmed that medical ethical approval is not required. We ensured that in our case studies, each sewer receives contributions from a minimum of 20 households/houses and findings could not be traced back to individuals, as suggested by Hrudey et al. [21]. Discussion about the ethical and juridical implications of sewage sampling are still ongoing in the Netherlands [22]. To address ethical concerns and facilitate early targeted deployment, engagement with local populations and close collaboration between public health, environmental and clinical stakeholders in advance or as early as possible in the process is recommended. Outcomes can be optimised: protocols should specify the necessity for sampling (such as outbreak response and health benefits); include strict criteria for data sharing (e.g. selective sharing of clinical data while ensuring patient anonymity); the timing and placement of passive samplers; sampling method to be applied and how results would be used.

A further limitation at the small area level is the daily fluctuations in people’s behaviour and virus shedding, water flow and viral concentration, which may lead to substantial variability (Figure 2). To control for this, technical duplicates and a relatively high measurement frequency (such as three 24-hour periods per week) is recommended for good coverage of short-term trends and early warning (and some robustness for missing data points). Where data needs were more semiquantitative, such as for the local transmission of Omicron or MPXV, the variability is less of a concern. In addition, boundaries of the wastewater catchment area were not always distinct and mixing from other sewage systems is possible. Detailed knowledge of the sewage system and catchment area is always key. Similarly, people travel for work or school from their residential area, so are not always represented via their residential sewers.

## Conclusion

Our study confirmed the feasibility and effectiveness of passive wastewater sampling for tracking the spread of SARS-CoV-2 and MPXV at the community level. We detected increased circulation of these viruses through surges in their nucleic acid concentrations in wastewater. This method provides a valuable tool for tracing outbreaks in a way that is simple and accessible. Passive samplers can be deployed rapidly and at many different places, yielding an agile, on-demand infectious disease surveillance system that can monitor at the desired resolution. Where the public health rationale for its application is clearly specified, agreed, and meets ethical guidelines, passive sampler technology has wide application in similar contexts across Europe where most of the population is linked to a sewage network. Despite our efforts to carefully match the area/population of the data obtained ‘above-ground’ (positive tests, vaccination rates, demographics) and ‘below-ground’ (wastewater monitoring), it is important to recognise that differences in resolution and areas may hamper a direct comparison. As the technology continues to evolve, it could be extended to other viruses, including respiratory viruses (e.g. influenza, respiratory syncytial virus) and enteric viruses (e.g. polio, entero, noro, hepatitis A and E virus), as well as bacteria (*Campylobacter,* Enterohaemorrhagic *Escherichia coli)* and antimicrobial resistance. Optimal stakeholder engagement and consideration of ethical and legal issues, especially on local sewage sampling, will be essential to ensure political and social acceptance.

## Supplementary Data

1236000 Supplementary Material
