# Supplementary material for: Agile, on-demand wastewater surveillance of virus infections to support pandemic and outbreak response in Rotterdam-Rijnmond, the Netherlands, 2020 to 2022
Source: Euro Surveill. 2024 Nov 21;29(47):2400055. doi: 10.2807/1560-7917.ES.2024.29.47.2400055 (PMC11583307; doi:10.2807/1560-7917.ES.2024.29.47.2400055)
Supplement: Supplementary Material [file 2400055_SupplementaryMaterial.pdf]

Supplementary information

This supplementary material is hosted by Eurosurveillance as supporting information alongside the article ‘Agile, on-demand wastewater surveillance of virus infections to support pandemic and outbreak response in Rotterdam-Rijnmond, the Netherlands, 2020 to 2022’ on behalf of the authors, who remain responsible for the accuracy and appropriateness of the content. The same standards for ethics, copyright, attributions and permissions as for the article apply. Supplements are not edited by Eurosurveillance and the journal is not responsible for the maintenance of any links or email addresses provided therein.

Table S.1 Evaluation of the wastewater surveillance case studies of Rotterdam Rijnmond against the attributes of a quality surveillance system.

Attributes	Case study 1	Case study 2	Case study 3	Case study 4	Case study 5	Case study 6
Objective	Passive sampling is a feasible method that could reliably detect SARS-CoV-2 in wastewater in different community contexts.	Evaluation of community transmission after the first case of the Omicron variant was identified in the region	Early warning of viral introduction in displaced populations.	Determine if student houses are hot-spots for introduction of SARS-CoV-2 in Rotterdam after the summer holidays	Determine if neighbourhoods with low vaccination coverage result in more SARS-CoV-2 infections	Detection of local transmission of MPXV
Sensitivity	Similar positivity rate and (normalized) concentrations of SARS-CoV-2 RNA in 24h volume-proportional samples and composite samples, similar concentration-trends over time observed in both sampler types.	Higher concentrations of SARS-CoV-2 were detected in round 1 (7-9 Dec 2021) than in round 2 (28-30 Dec 2021). No Omicron was identified during week 1 (ddPCR and sequencing) but its presence was confirmed in week 2 in both passives and sewage water. Numerical values of the Omicron proportion were variable, probably due to relatively low proportions of Omicron.	Due to potential stigmatization of this population, and the complexity of obtaining consent from all stakeholders the case study was cancelled.	SARS-CoV-2 was detected at each student home, but at different positivity rates and (normalised) concentrations. CrAssphage indicated similar amounts of human faeces were sampled in each sample.	Virus circulation was shown amongst all the populations measured. Distribution of viral circulation was equal in most neighbourhoods, except for one of the neighbourhoods with high vaccine coverage, which had a high amount of SARS-CoV-2 present in October 2022. There was no explanation for why this area had higher virus-circulation.	MPXV was found in the sewage water of the area in 20-80 gene copies per passive sample in the wastewater in the street of a known case. The presence of MPXV was shown in the sewage water, in low copy numbers in 2 samples, which were positive for both the Orthopox qPCR and MPXV qPCR and ddPCR assay.
Representativeness	A large COVID-19 peak was detected both in clinical data from the last week of October and in wastewater tested on 27 of October 2021. In areas with low circulation, trends were more variable. The overall trend observed with passive samplers was similar to the	Omicron BA.1 was detected in round 2 at each site in the area		The student houses surveyed were representative of larger student residences, common in the Netherlands, that house 80-150 students in single-person studios, or shared apartments within the same building.	All neighbourhoods were adequately measured, with the passives distributed across 8 sewer pumping stations to cover the complete neighbourhoods with all inhabitants. One neighbourhood needed to be sampled at 3 pumping stations, one at 2 and the	MPXV was found in the wastewater in the street of a known case.

	trend observed with a 24h volume-proportional sampler.				other 3 at one pumping station each.	
Completeness	Sites were selected from the sewer network to cover the different city area populations and matched with the clinical surveillance via zip code. Deployment of passive samplers was feasible at each selected site.	Sites were selected from the sewer network to cover the area of the case residence and the wider community via zip code. Deployment of passive samplers was feasible at each selected site and 24h volume composite samples were also available for the wastewater treatment plant. This allowed comparison between passive and composite samplers.		Proof of concept, using 5 student houses as sentinel. Sites were selected from the sewer network to cover the 5 student houses. Deployment of passive samplers was feasible at each selected site.	Sites were selected from the sewer network to cover the city areas with different vaccination rates. Deployment of passive samplers was feasible at each selected site. All identified neighbourhoods were sampled for 2.5 months.	Sites were selected from the sewer network to cover the area of the case residence and the wider community via zip code. Proof of concept: shown for only one local case. Repeated for a second case (data not shown).
Validity	4/80 passives could not be recovered from the sampling site (3 as the suspending strings became entwined due to variable water flow and the strings broke, 1 for reasons unknown). Passives were no longer installed in parallel & no more went missing. 72/76 yielded a valid result. 4 results were considered invalid because CrAssphage	All samples were successfully recovered. The recovery was high for all passives. Duplicate wastewater samples showed very little variation. The concentrations in passive samples correlated well (after normalization) with 24-hour volume-proportional wastewater samples.		134 of 140 samples provided valid results. The SARS-CoV-2 PCR control indicated lab errors in 4 samples. The CrAssphage assay indicated 2 samplers had captured very little human faecal material.	229/238 passive samples produced a valid result. 1 passive was lost because the rope was cut, 1 did not contain cotton tips. SARS-CoV-2 PCR control indicated that 7 samples did not produce a valid result due to lab errors.	All 9 passive samplers that were deployed produced valid results. Quality controls including positive and negative PCR controls, blank samples and PCR inhibition controls, gave good results. Combining RT-PCR assays of both Orthopox and MPXV assays as well as an additional ddPCR assay for West African MPXV provided confirmation. CrAssphage

	indicated very low recovery of human faecal material on the passive sampler. The other passive sampler results were reliable and correlated well with 24-hour sewage samples. Triplicates, measured daily for 1 week, provided full, valid results on 3 days and agreed well with 24 hour-sampling (they deviated substantially on 1 day). Averaged results from passives compared well with results from the pumping station on 4/5 days but deviated by a factor of 3 on day 5.					was used to determine amount of human faecal material captured on the passive samplers, as previously described.
Timeliness	Although not critical here, appropriate sampling sites in the sewer network were identified within a week. Sites were sampled at the planned timing.	Passives reached and were processed in the lab on the same day as they were recollected from the test location. N2 and CrAssphage measurements were available in 1 day. Results were available within 5 days after the request the ddRT-PCR results on day 6 & sequencing on day 14.		Preplanned 2 months before start. The samples of a week were analysed as one batch, making the time-to-result 1-5 days.	Preplanned 2 months before start. The samples of a week were analysed as one batch, making the time-to-result 1-5 days.	Implementation was executed within days of the availability of information surrounding the first cases in Rotterdam-Rijnmond. The first series of passive samplers were installed in the sewer mains that drained the area of the mpox case residences within 2 days of receipt of the request. The samples of the week were analysed as one batch, making the time-to-result 1-5 days.

Acceptability	The city areas were large enough for the sampling to be anonymous, but it was recognized that smaller areas could result in potential identification. Also the potential for stigmatization was indicated.	Even though this was at a small scale, the area of the case was sampled without the address disclosed, and with sufficient other addresses to ensure the case was not identifiable.		Letters were sent out to the housing organisations of the student locations to inform them of the research. They were facilitated to easily inform the residents. As described in the main text, informed consent was not required.	Areas were sufficiently large not to result identifiable cases.	Even though this was at a small scale, the area of the case was sampled without the address disclosed, and with sufficient other addresses to ensure the case was not identifiable.
Simplicity	All sites were readily identified and could be sampled without obstructing traffic. Processing of passive samples in the lab was conducted without problems.	Within 1 hour and 45 minutes of request receipt, the above-ground location was matched to the underground sewer infrastructure. The first passive was installed on the next working day.		Samplers were readily deployed to selected manholes. One manhole was not reachable at sampling day 10, due to construction work. All samplers were recovered.	Identification of appropriate sampling sites in areas of different vaccination rates was rapid. Passive samplers were easy to deploy at sewer pumping stations, only 1 of 238 samplers was lost.	Manholes were readily identified and sampled in the sewer network in the area of the case (without disclosing the address, and with sufficient other addresses to ensure the case was not identifiable).
Flexibility	All desired locations were sampled, both via manholes and pumping stations, and reference samples were collected at the pumping stations at the same time.	Samples could be taken close to the case, as well as a larger surrounding area via a pumping station, and a larger area via the wastewater treatment plant		Student homes sampled in planned period.	All desired locations in the different city areas were sampled.	All desired locations sampled.
Consistency	At small (6600-21000) population scale daily fluctuations were substantial and trend extraction was more difficult and less reliable than observed in earlier research at larger population scale (>75,000-128,000).	Not evaluated as only 1 case was reported at the time in this region		The three student locations showed different and dynamic patterns of SARS-CoV-2 concentrations in their wastewater, also after normalization for CrAssphage. The concentration in student homes was relatively low compared to the city areas and overall city wastewater data, indicating the virus was	The different city areas showed dynamic patterns of SARS-CoV-2 concentrations in their wastewater, also after normalization for CrAssphage. High variation per site hampered trend detection. One of the areas with high vaccination coverage showed a clear peak in concentrations in October 2022, so more prominent	Not evaluated.

				not particularly circulation among students at the time of the study.	trends were more discernible.	
Usefulness	Definition of minimal time from request to result: 3 days, but only if site selection is fast. A large COVID-19 peak was detected both in clinical data from the last week of October and in wastewater tested on 27 of October 2021.	Samples processed from passives could be sequenced just as well as from sewage water samples. Omicron was identified through ddPCR and sequencing in round 2 of testing at each of the sampling sites, indicating the VOC had spread through the community and quarantining and contact tracing was not a feasible option to contain further spread.		Variability of the observed concentration in sewers from the student houses obscured firm conclusions about the students as 'introducers' after the summer holidays.	Results were different than expected, and appear not to align with reports that vaccination protects contacts from infection. But differences in vaccination coverage are not very large and no other information on frequency and diversity of contacts of the communities is available.	The presence in the wastewater in the street of the case, but not in the surrounding area, showed proof of concept of MPXV detection in wastewater and supported no further local spread of MPXV.

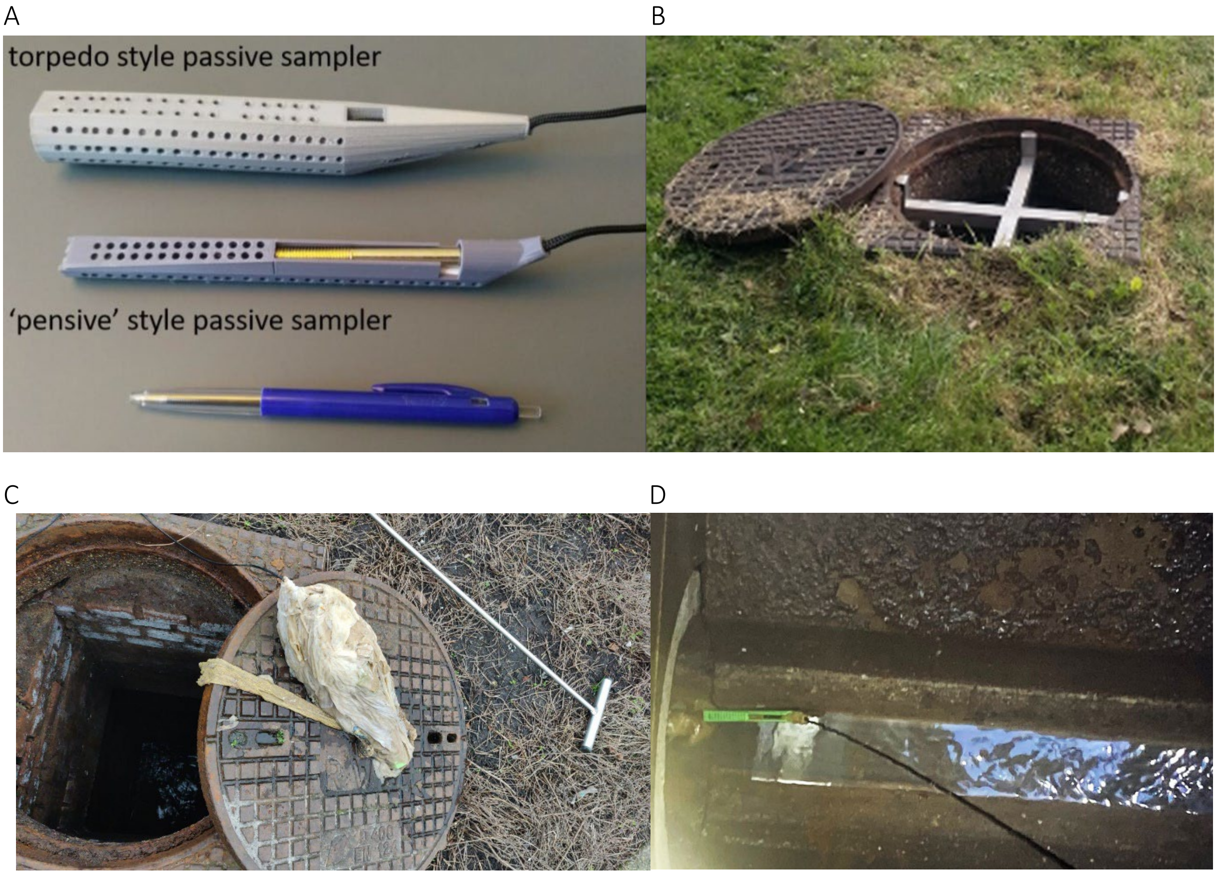

Figure S.1 Passive and pensive sampler (A) and installation in manhole (B) and of the quality control of the samplers, showing large amounts of wipes deposited on the sampler (C) or the sampler moved outside of the wastewater flow (D).

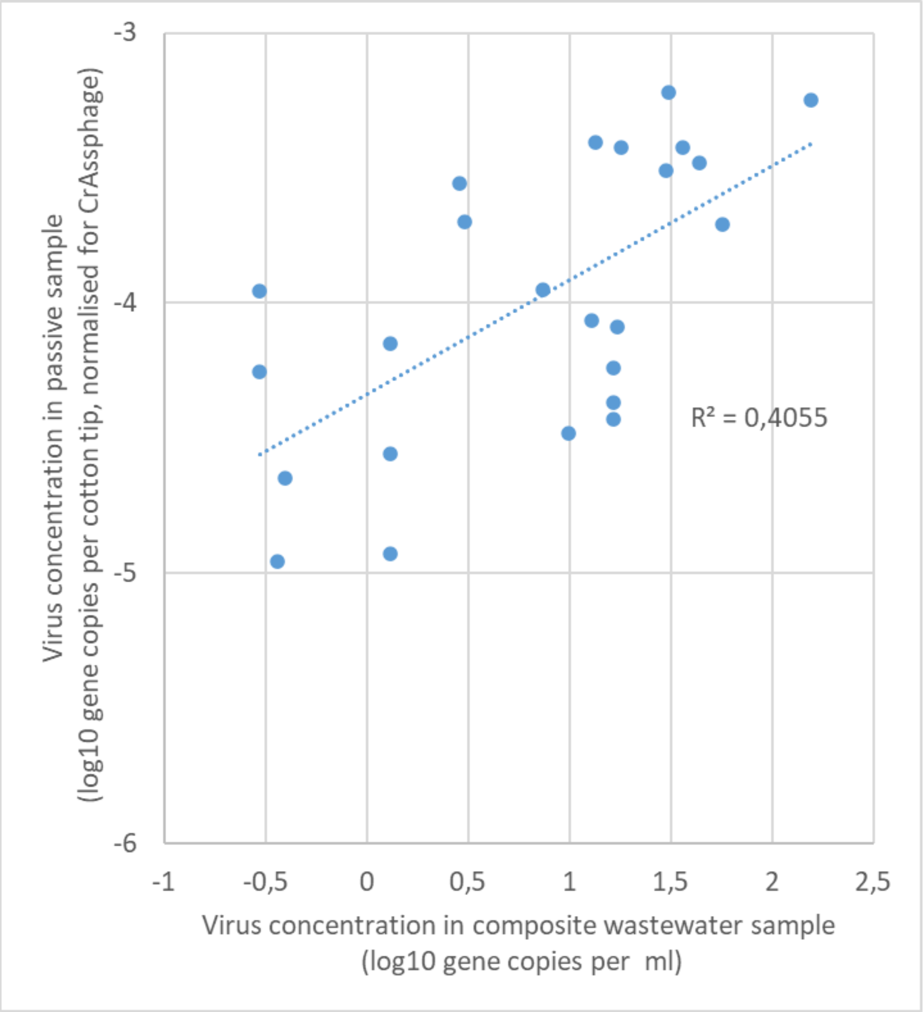

Figure S.2. Correlation between SARS-CoV-2 concentrations from 24-hour the passive samplers, normalised for CrAssphage (Y-axis) and the 24-hour volume-proportional wastewater samples for virus capture from wastewater (X-axis).

Left

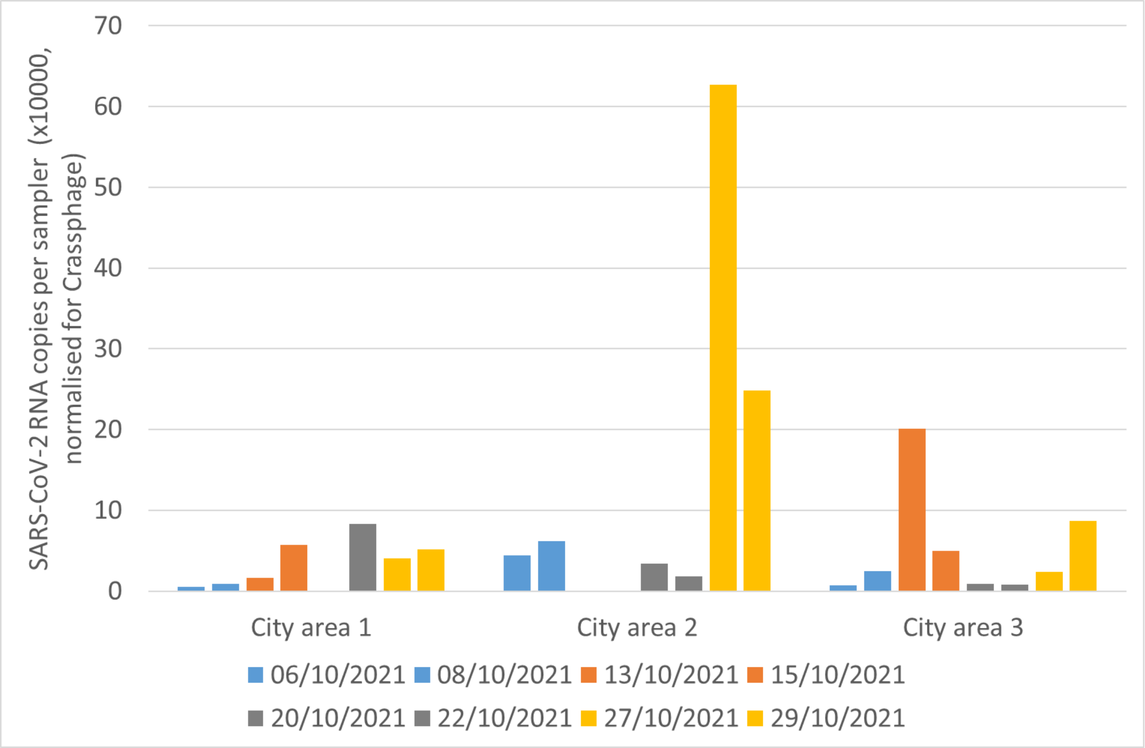

Right

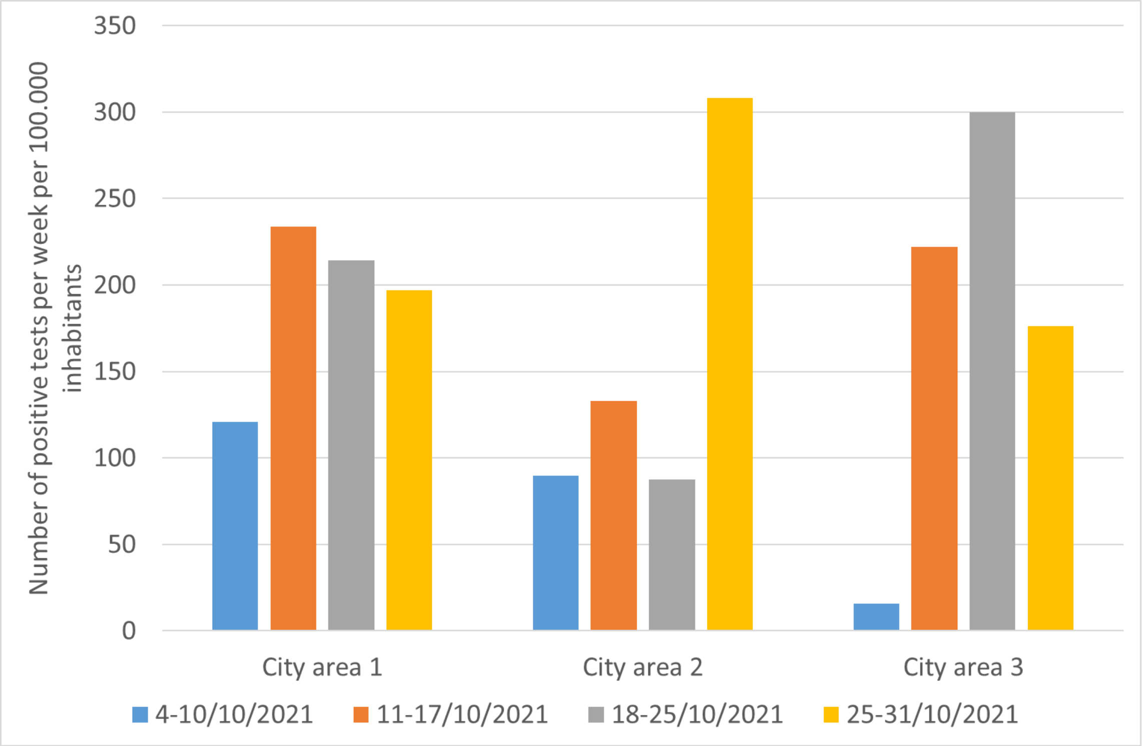

Figure S.3 Dynamics of the wastewater concentration of SARS-CoV-2 with passive samplers in small city areas (normalised for Crassphage, left) and the positive cases reported in the same areas and weeks (right). Two passive samples were taken each week, each sampling for 24 hours. The number of positive cases was reported on a weekly basis and the data reported in the week that encompassed the dates of the wastewater samples were used (see the corresponding colours in the left and right graph). In city area 2, no SARS-CoV-2 RNA was detected in the samples of 13 and 15 Octobre 2021. In

city area 1, SARS-CoV-2 RNA was detected in the sample of Octobre 13, 2021, but the Crassphage analysis was not reliable, so no normalized concentration could be calculated.

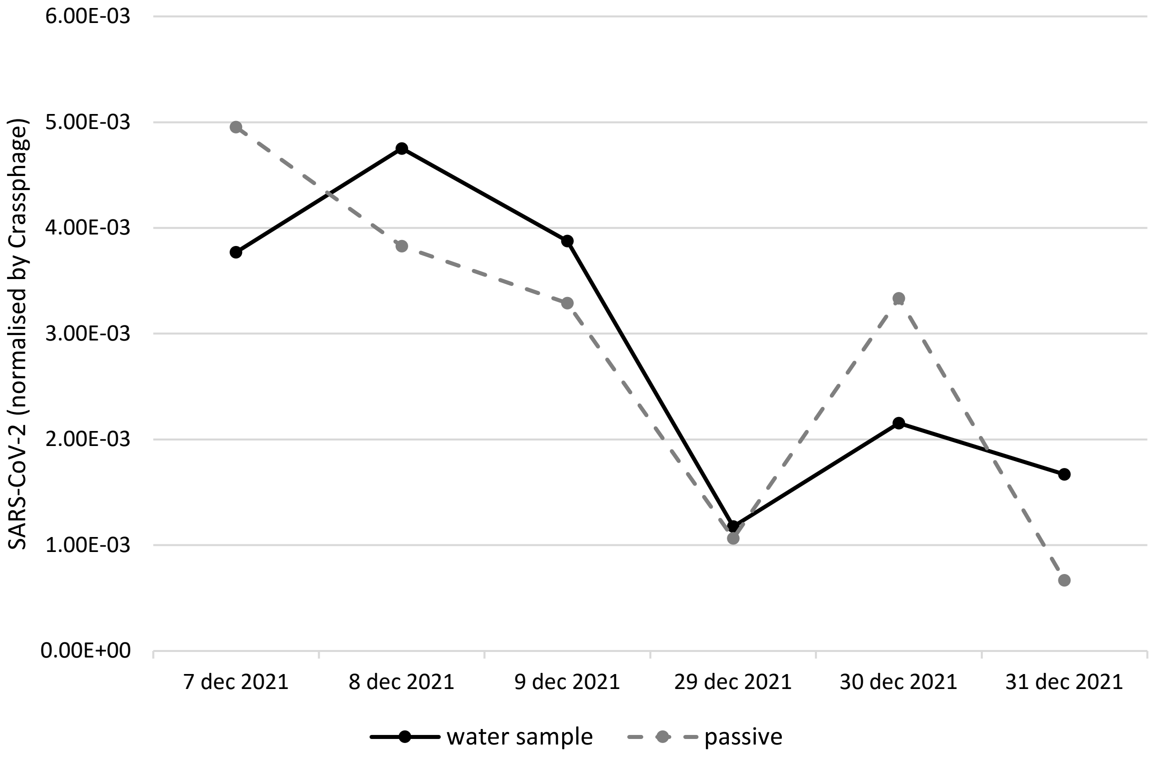

Figure S.4. Comparison of normalized SARS-CoV-2 RNA concentrations (SARS-CoV-2 gc/ml divided by Crassphage GC/ml) in 24-hour water sampling versus in passive samples in Case Study 2, detection of omicron variant in wastewater after the first diagnosis in the region, Rotterdam-Rijnmond, the Netherlands, December 2021.

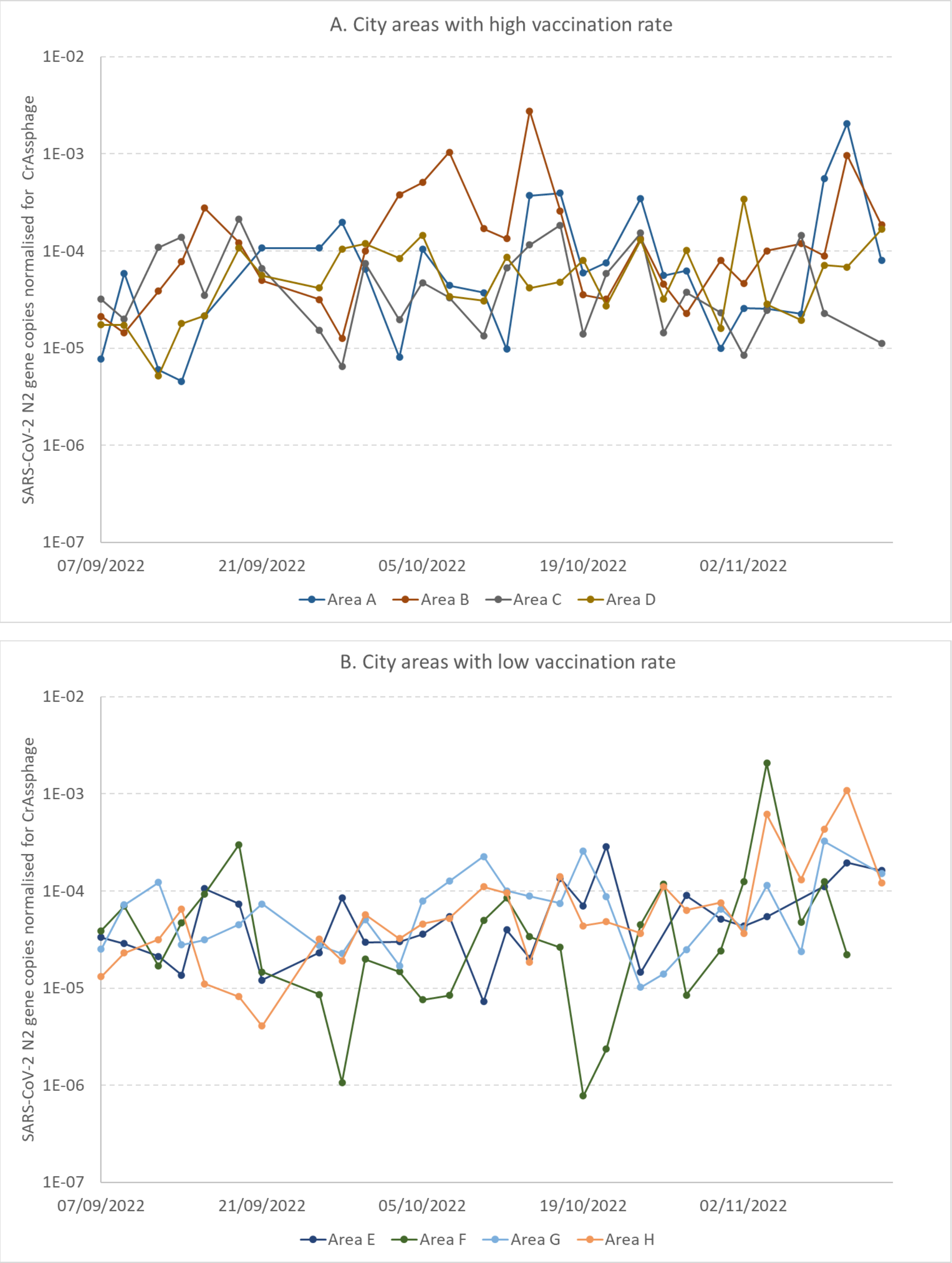

Figure S.5. Concentration of SARS-CoV-2 RNA in wastewater (normalized for CrAssphage) in city areas with high (A) and low (B) vaccination rates in September – November 2022.

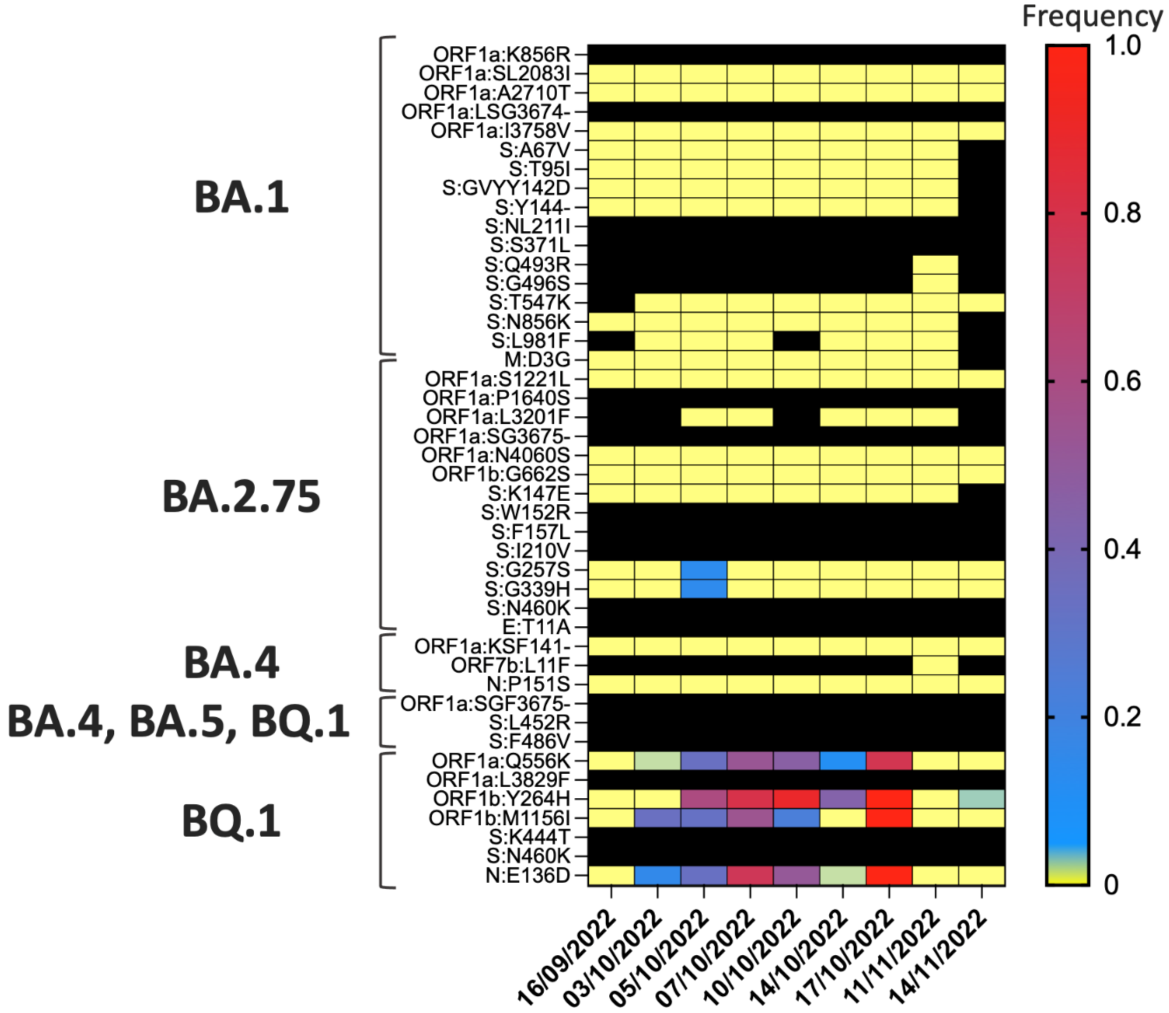

Figure S.6. Relative abundance of SARS-CoV-2 major lineages in the wastewater samples in Case study 5. Heatmap showing the frequency of the unique signature mutations per lineage.
